# Supplementary material for: The Evolution and Characterization of the RNA Interference Pathways in Lophotrochozoa
Source: Genome Biol Evol. 2024 May 7;16(5):evae098. doi: 10.1093/gbe/evae098 (PMC11114477; doi:10.1093/gbe/evae098)
Supplement: evae098_Supplementary_Data [file evae098_supplementary_data.zip › Supplementary_materials.zip!/selected_miRNAs/NoGe_selected_miRNAs/mirdeep_pdf_files/NMRB01000102.1_Notospermus_geniculatus_strain_Ushimado_scaffold102,_whole_genome_shotgun_sequence_3913.pdf]

Provisional ID	:	NMRB01000102.1_Notospermus_geniculatus_strain_Ushimado_scaffold102,_whole_genome_shotgun_sequence_3913
Score total	:	54451.6
Score for star read(s)	:	3.9
Score for read counts	:	54446.4
Score for mfe	:	1.9
Score for randfold	:	0
Score for cons. seed	:	-0.6
Total read count	:	106806
Mature read count	:	106572
Loop read count	:	0
Star read count	:	234

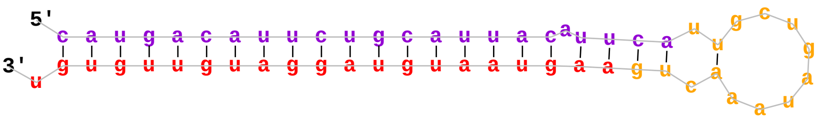

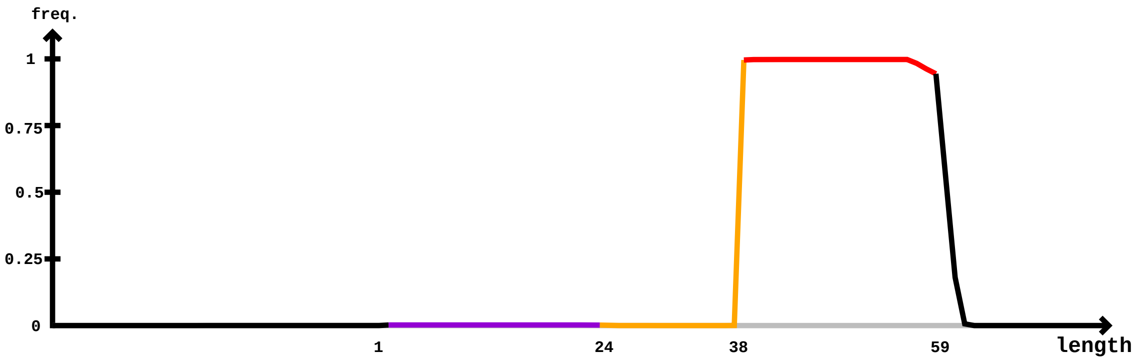

Star **Mature**

```
5'- gaagguugaaggaauacgcuccuucugaugggacaugacauucugcauuacauucauugcugauaaacugaaguaauguaggauguuguguuucaugagaaaggaagg  -3'   obs
    gaagguugaaggaauacgcuccuucugaugggacaugacauucugcauuacauucauugcugauaaacugaaguaauguaggauguuguguuucaugagaaaggaagg         exp
    ...................(((((((.((((((((((((((((((((((((.((((.(........).)))))))))))))))))))))))))))).)).))))).  reads  mm  sample
    ..............uacgcuccuucugauggga...........................................................................  1  0  Mm3
    .................................caugacauucugcauuacauu......................................................  1  0  Mm3
    .................................caugacauucugcauuacauuc.....................................................  4  0  Mm3
    .................................caugacauucugcauuaUauuca....................................................  1  1  Mm3
    .................................caugacauucugcauuacauuca....................................................  40  0  Mm3
    .................................caugacauucugcauuacauucaA...................................................  1  1  Mm3
    .................................caugacauucugcauuacauucauugc................................................  1  0  Mm3
    .................................caugacauucugcauuacauucauugcu...............................................  7  0  Mm3
    .................................caugacauucugcauuacauucauugcug..............................................  1  0  Mm3
    ......................................................................aaguaauguaggauguuU....................  1  1  Mm3
    ......................................................................aaguaauguaggauguugu...................  29  0  Mm3
    ......................................................................aaguaauguaggauguuAug..................  1  1  Mm3
    ......................................................................aaguaauguaggauguuguA..................  1  1  Mm3
    ......................................................................aaguaauguaggauguugug..................  6  0  Mm3
    ......................................................................aaguaauguaggauguuguU..................  5  1  Mm3
    ......................................................................aaguaauguaggauguuAugu.................  1  1  Mm3
    ......................................................................aaguaauguaUgauguugugu.................  1  1  Mm3
    ......................................................................aaguaauguaggauguuguAu.................  1  1  Mm3
    ......................................................................aaguaauguaggauguugugu.................  463  0  Mm3
    ......................................................................aaguaauAuaggauguugugu.................  1  1  Mm3
    ......................................................................aaguaauguaggauguuUugu.................  1  1  Mm3
    ......................................................................aagCaauguaggauguugugu.................  2  1  Mm3
    ......................................................................Uaguaauguaggauguugugu.................  2  1  Mm3
    ......................................................................aaguaauguaggauguugugA.................  2  1  Mm3
    ......................................................................aaguaauguaggauguuCugu.................  2  1  Mm3
    ......................................................................aaguaaugCaggauguugugu.................  1  1  Mm3
    ......................................................................aaguaauguaggauguCgugu.................  1  1  Mm3
    ......................................................................aaguaauguaggauguugugG.................  45  1  Mm3
    ......................................................................aaguaauguaggUuguuguguu................  1  1  Mm3
    ......................................................................aaguaaAguaggauguuguguu................  1  1  Mm3
    ......................................................................aaguaauguaggauguugugGu................  1  1  Mm3
    ......................................................................aaguaGuguaggauguuguguu................  1  1  Mm3
    ......................................................................aaguaauguaggauguuguAuu................  1  1  Mm3
```

```
                                   Star                                   Mature
gaagguugaaggaauacgcuccuucugaugggacaugacauucugcauuacauucauugcugauaaacugaaguaauguaggauguuguguuucaugagaaaggaagg
......................................................................aagCaauguaggauguuguguu................   1      1   Mm3
......................................................................aaguaUuguaggauguuguguu................   2      1   Mm3
......................................................................aaguaauguaAgauguuguguu................   8      1   Mm3
......................................................................aaguaauguaCgauguuguguu................   2      1   Mm3
......................................................................aaguaauguaggauguGguguu................   7      1   Mm3
......................................................................aaguUauguaggauguuguguu................   1      1   Mm3
......................................................................Gaguaauguaggauguuguguu................   2      1   Mm3
......................................................................aaguaauguaggauguuguguC................   2      1   Mm3
......................................................................aUguaauguaggauguuguguu................   3      1   Mm3
......................................................................aaguaauguGggauguuguguu................   2      1   Mm3
......................................................................aGguaauguaggauguuguguu................   1      1   Mm3
......................................................................aaguaauguaggauguAguguu................   9      1   Mm3
......................................................................aaguaaugAaggauguuguguu................   1      1   Mm3
......................................................................aaguaauguaggauguuguguu................   4339   0   Mm3
......................................................................aaguaauguaggGuguuguguu................   2      1   Mm3
......................................................................Caguaauguaggauguuguguu................   2      1   Mm3
......................................................................aaguaaugCaggauguuguguu................   2      1   Mm3
......................................................................aagAaauguaggauguuguguu................   1      1   Mm3
......................................................................aaguaauguUggauguuguguu................   1      1   Mm3
......................................................................aaguaauguaggauguugugCu................   1      1   Mm3
......................................................................aaguaauguaggauguuguguG................   8      1   Mm3
......................................................................aaguaauguaggauguuCuguu................   27     1   Mm3
......................................................................aaguaauguaggauguuUuguu................   20     1   Mm3
......................................................................aaguaauguaggauguuAuguu................   30     1   Mm3
......................................................................Uaguaauguaggauguuguguu................   6      1   Mm3
......................................................................aaguaauguaggauguuguguA................   10     1   Mm3
......................................................................aaguaauguaggauguCguguu................   20     1   Mm3
......................................................................aaguaNuguaggauguuguguu................   1      1   Mm3
......................................................................aaguaauguaggaCguuguguu................   1      1   Mm3
......................................................................aagAaauguaggauguuguguuu...............   3      1   Mm3
......................................................................aaguaauguaggauguCguguuu...............   5      1   Mm3
......................................................................Uaguaauguaggauguuguguuu...............   1      1   Mm3
......................................................................aGguaauguaggauguuguguuu...............   1      1   Mm3
......................................................................aCguaauguaggauguuguguuu...............   1      1   Mm3
......................................................................aaguaauguaggauguuguguAu...............   5      1   Mm3
......................................................................aaguaauguaggauguuAuguuu...............   7      1   Mm3
......................................................................Caguaauguaggauguuguguuu...............   1      1   Mm3
......................................................................aaguaaCguaggauguuguguuu...............   1      1   Mm3
......................................................................aaguaauguaCgauguuguguuu...............   2      1   Mm3
......................................................................aaguaauguaggauguuUuguuu...............   6      1   Mm3
......................................................................aaguaauguaggauguuguguuu...............   1567   0   Mm3
......................................................................aaguaauguaggauguuCuguuu...............   10     1   Mm3
......................................................................aaguaauguaggauguAguguuu...............   9      1   Mm3
......................................................................Naguaauguaggauguuguguuu...............   1      1   Mm3
......................................................................aaguaauguaggauguuguguuG...............   7      1   Mm3
......................................................................aaguaauguaggUuguuguguuu...............   3      1   Mm3
......................................................................aaguaaGguaggauguuguguuu...............   1      1   Mm3
......................................................................aaguaauguaAgauguuguguuu...............   3      1   Mm3
......................................................................aaguaauguaggauguuguguuA...............   136    1   Mm3
......................................................................aaguaauguaggauguuguguuC...............   5      1   Mm3
......................................................................aaguaauguaggauguGguguuu...............   1      1   Mm3
......................................................................aaUuaauguaggauguuguguuu...............   1      1   Mm3
......................................................................Gaguaauguaggauguuguguuu...............   1      1   Mm3
......................................................................aaguaUuguaggauguuguguuu...............   1      1   Mm3
......................................................................aaguaauguaggauguuguguuuU..............   71     1   Mm3
......................................................................aaguaauguaggauguuguguuuA..............   3      1   Mm3
......................................................................aaguaauguaggauguuguguuuG..............   1      1   Mm3
.......................................................................aguaauguaggauguuguA..................   2      1   Mm3
.......................................................................aguaauguaggauguugugu.................   3      0   Mm3
.......................................................................aguaauguUggauguuguguu................   1      1   Mm3
.......................................................................aguaauguagAauguuguguu................   1      1   Mm3
.......................................................................aguaaAguaggauguuguguu................   1      1   Mm3
.......................................................................aguaauguaggauguuguguu................   5      0   Mm3
.......................................................................aguaauguaggauguuguguuuU..............   1      1   Mm3
.........................................................................uaauguaggauguuguguu................   1      0   Mm3

..............uacgcuccuucugauggga...........................................................................   7      0   Fm3
.................................Uaugacauucugcauuacauu......................................................   1      1   Fm3
.................................caugacauucugcauuacauu......................................................   11     0   Fm3
.................................caugacauucugcauuacauuc.....................................................   5      0   Fm3
```

Star **Mature**

```
gaagguugaaggaauacgcuccuucugaugggacaugacauucugcauuacauucauugcugauaaacugaaguaauguaggauguuguguuucaugagaaaggaagg
.................................caugacauucugcauuaAauuca....................................................   1     1   Fm3
.................................caugacauucugcauuacauuca....................................................   35    0   Fm3
.................................caugacauucugcauuacauucauugcu...............................................   2     0   Fm3
.....................................................................Caaguaauguaggauguugugu.................   3     1   Fm3
.....................................................................Caaguaauguaggauguuguguu................   16    1   Fm3
......................................................................aaguaauguaggauguug....................   1446  0   Fm3
......................................................................Uaguaauguaggauguug....................   5     1   Fm3
......................................................................aaguaauguaggauguAg....................   9     1   Fm3
......................................................................aaguaauguaggauguuC....................   9     1   Fm3
......................................................................aaguaauguaggauUuug....................   1     1   Fm3
......................................................................aaCuaauguaggauguug....................   1     1   Fm3
......................................................................aaguGauguaggauguug....................   1     1   Fm3
......................................................................Naguaauguaggauguug....................   1     1   Fm3
......................................................................aaguaauguaAgauguug....................   2     1   Fm3
......................................................................aaguaauguaggauguuA....................   21    1   Fm3
......................................................................aaguaauguaggauguuU....................   11    1   Fm3
......................................................................aaguaauguaCgauguug....................   1     1   Fm3
......................................................................aaguaauguaggauguCg....................   6     1   Fm3
......................................................................aaguaauguaggauguGg....................   5     1   Fm3
......................................................................aaguCauguaggauguug....................   1     1   Fm3
......................................................................aaguaauguaUgauguug....................   1     1   Fm3
......................................................................aaguaaugAaggauguug....................   2     1   Fm3
......................................................................aaguaauguaggaAguugu...................   2     1   Fm3
......................................................................aaguaauguGggauguugu...................   1     1   Fm3
......................................................................aaguaauguaggauguugA...................   84    1   Fm3
......................................................................aaguaauguaggauguugC...................   4     1   Fm3
......................................................................aaguaaGguaggauguugu...................   1     1   Fm3
......................................................................aaguaauguaggauguuUu...................   14    1   Fm3
......................................................................aaguaauguaggauguCgu...................   7     1   Fm3
......................................................................aaguaauguaggauguuAu...................   13    1   Fm3
......................................................................aaguaauguaggauguGgu...................   5     1   Fm3
......................................................................aaguaauguaggauguugu...................   1791  0   Fm3
......................................................................Naguaauguaggauguugu...................   1     1   Fm3
......................................................................aGguaauguaggauguugu...................   1     1   Fm3
......................................................................aaguaaugAaggauguugu...................   1     1   Fm3
......................................................................aUguaauguaggauguugu...................   2     1   Fm3
......................................................................aaguaauguaUgauguugu...................   2     1   Fm3
......................................................................aaguaauguaggauguuCu...................   6     1   Fm3
......................................................................aaguaauguaggUuguugu...................   1     1   Fm3
......................................................................aaguaauguaAgauguugu...................   4     1   Fm3
......................................................................aaguaCuguaggauguugu...................   1     1   Fm3
......................................................................Uaguaauguaggauguugu...................   4     1   Fm3
......................................................................Gaguaauguaggauguugu...................   1     1   Fm3
......................................................................aaguaauguaggauguAgu...................   2     1   Fm3
......................................................................aaNuaauguaggauguugug..................   1     1   Fm3
......................................................................Caguaauguaggauguugug..................   1     1   Fm3
......................................................................aaguaauguaggauguuguU..................   44    1   Fm3
......................................................................aaguaaNguaggauguugug..................   1     1   Fm3
......................................................................aagAaauguaggauguugug..................   1     1   Fm3
......................................................................aaguaauguaggauguCgug..................   3     1   Fm3
......................................................................aaguaauguaUgauguugug..................   2     1   Fm3
......................................................................aaguaauguaAgauguugug..................   2     1   Fm3
......................................................................aaguaauguaggauguuUug..................   4     1   Fm3
......................................................................aaguaauguCggauguugug..................   1     1   Fm3
......................................................................aaguaauguaggauguAgug..................   3     1   Fm3
......................................................................aaguaauguaggauguuCug..................   4     1   Fm3
......................................................................aaguaauguaggauguuguC..................   1     1   Fm3
......................................................................aaguaauguaggauguGgug..................   4     1   Fm3
......................................................................aaguaauguaggauguuAug..................   6     1   Fm3
......................................................................aaguaauguaggauguugug..................   1641  0   Fm3
......................................................................aaguaaugAaggauguugug..................   1     1   Fm3
......................................................................Uaguaauguaggauguugug..................   3     1   Fm3
......................................................................aUguaauguaggauguugug..................   1     1   Fm3
......................................................................aaguaauguaggauguuguA..................   8     1   Fm3
......................................................................Naguaauguaggauguugug..................   1     1   Fm3
......................................................................aaguaCuguaggauguugugu.................   2     1   Fm3
......................................................................aaguaauguagAauguugugu.................   2     1   Fm3
......................................................................aaguaauguaggaugGugugu.................   1     1   Fm3
......................................................................aCguaauguaggauguugugu.................   7     1   Fm3
......................................................................aaguaauUuaggauguugugu.................   4     1   Fm3
```

```
                                 Star                                  Mature
gaagguugaaggaauacgcuccuucugauggga caugacauucugcauuacauuca uugcugauaaacug aaguaauguaggauguugugu uucaugagaaaggaagg
......................................................................aaguaauguaggauguuguAu.................   7       1   Fm3
......................................................................aaguaauguUggauguugugu.................   4       1   Fm3
......................................................................aaUuaauguaggauguugugu.................   6       1   Fm3
......................................................................aaguGauguaggauguugugu.................   18      1   Fm3
......................................................................aaguaauguaggaugCugugu.................   6       1   Fm3
......................................................................aaAuaauguaggauguugugu.................   6       1   Fm3
......................................................................Gaguaauguaggauguugugu.................   5       1   Fm3
......................................................................aaguaauguaggauguugAgu.................   5       1   Fm3
......................................................................aagAaauguaggauguugugu.................   16      1   Fm3
......................................................................aaguaaNguaggauguugugu.................   1       1   Fm3
......................................................................aaguaauguaggauguuCugu.................   169     1   Fm3
......................................................................aaguaauguaggauCuugugu.................   3       1   Fm3
......................................................................aaguaauguaggauguugugC.................   3       1   Fm3
......................................................................aaguaauguaggauguuNugu.................   1       1   Fm3
......................................................................Caguaauguaggauguugugu.................   7       1   Fm3
......................................................................aaguaauguaggaugAugugu.................   3       1   Fm3
......................................................................aaguaauguaggauguGgugu.................   92      1   Fm3
......................................................................aaguaaugGaggauguugugu.................   1       1   Fm3
......................................................................aaCuaauguaggauguugugu.................   1       1   Fm3
......................................................................Naguaauguaggauguugugu.................   17      1   Fm3
......................................................................aaguaauguaggaAguugugu.................   6       1   Fm3
......................................................................aaguaauguaggaGguugugu.................   2       1   Fm3
......................................................................aaguaauguGggauguugugu.................   2       1   Fm3
......................................................................aaguaauguaggauguugugu.................   34539   0   Fm3
......................................................................aaguaaugAaggauguugugu.................   6       1   Fm3
......................................................................aagCaauguaggauguugugu.................   17      1   Fm3
......................................................................aaguaauguaggauguuAugu.................   177     1   Fm3
......................................................................aaguaauguCggauguugugu.................   2       1   Fm3
......................................................................aaguaauguaggaCguugugu.................   1       1   Fm3
......................................................................aaguaauguaggauguAgugu.................   71      1   Fm3
......................................................................aaguaauguaggauAuugugu.................   2       1   Fm3
......................................................................aagGaauguaggauguugugu.................   6       1   Fm3
......................................................................aaguaauguaggauguuUugu.................   146     1   Fm3
......................................................................aaguaauguaggauguuguUu.................   22      1   Fm3
......................................................................aaguaauguaCgauguugugu.................   23      1   Fm3
......................................................................aaguaaugCaggauguugugu.................   7       1   Fm3
......................................................................aaguaauAuaggauguugugu.................   4       1   Fm3
......................................................................aGguaauguaggauguugugu.................   17      1   Fm3
......................................................................aaguaauguaggauguugCgu.................   3       1   Fm3
......................................................................aaguaauguaggauguugugA.................   136     1   Fm3
......................................................................aaguaaCguaggauguugugu.................   8       1   Fm3
......................................................................aaguaauguaggauguugugG.................   1       1   Fm3
......................................................................aaguaauguaAgauguugugu.................   57      1   Fm3
......................................................................aaguaauguagUauguugugu.................   8       1   Fm3
......................................................................aaguCauguaggauguugugu.................   3       1   Fm3
......................................................................aaguaauguaUgauguugugu.................   44      1   Fm3
......................................................................Uaguaauguaggauguugugu.................   60      1   Fm3
......................................................................aaguaauguaggauguCgugu.................   114     1   Fm3
......................................................................aaguaNuguaggauguugugu.................   4       1   Fm3
......................................................................aaguaUuguaggauguugugu.................   12      1   Fm3
......................................................................aaguaaAguaggauguugugu.................   3       1   Fm3
......................................................................aaguUauguaggauguugugu.................   2       1   Fm3
......................................................................aaguaauguaggCuguugugu.................   1       1   Fm3
......................................................................aaguaaGguaggauguugugu.................   1       1   Fm3
......................................................................aaguaauguaggGuguugugu.................   3       1   Fm3
......................................................................aaguaauguaggUuguugugu.................   3       1   Fm3
......................................................................aaguaGuguaggauguugugu.................   4       1   Fm3
......................................................................aUguaauguaggauguugugu.................   16      1   Fm3
......................................................................aaguaauguaggauUuugugu.................   3       1   Fm3
......................................................................aaguaauguUggauguuguguu................   2       1   Fm3
......................................................................aaguaauguagUauguuguguu................   4       1   Fm3
......................................................................aaguaGuguaggauguuguguu................   2       1   Fm3
......................................................................Caguaauguaggauguuguguu................   3       1   Fm3
......................................................................aUguaauguaggauguuguguu................   6       1   Fm3
......................................................................aaguaauguGggauguuguguu................   3       1   Fm3
......................................................................aaguaauguaggauAuuguguu................   3       1   Fm3
......................................................................aaguaauguaggauguuAuguu................   89      1   Fm3
......................................................................aaguaauguagAauguuguguu................   1       1   Fm3
......................................................................aaguaauguaggauguuguUuu................   4       1   Fm3
......................................................................aaguaauguaAgauguuguguu................   29      1   Fm3
```

```
                                Star                                Mature
gaagguugaaggaauacgcuccuucugaugggacaugacauucugcauuacauucauugcugauaaacugaaguaauguaggauguuguguuucaugagaaaggaagg
......................................................................aagCaauguaggauguuguguu................    5      1    Fm3
......................................................................aaguaauguaggaugGuguguu................    1      1    Fm3
......................................................................aaguaauguaggauguugugAu................    4      1    Fm3
......................................................................aaCuaauguaggauguuguguu................    1      1    Fm3
......................................................................aaguaaugGaggauguuguguu................    1      1    Fm3
......................................................................aagAaauguaggauguuguguu................    5      1    Fm3
......................................................................aaguaauguaggauguAguguu................    49     1    Fm3
......................................................................aaguaauguaggauCuuguguu................    1      1    Fm3
......................................................................aaguaauguaggCuguuguguu................    2      1    Fm3
......................................................................aaguaauAuaggauguuguguu................    1      1    Fm3
......................................................................aaguaauguaggauguuguguC................    2      1    Fm3
......................................................................aaguaNuguaggauguuguguu................    1      1    Fm3
......................................................................Uaguaauguaggauguuguguu................    31     1    Fm3
......................................................................aaguaauguaggauguuguguA................    10     1    Fm3
......................................................................aaguCauguaggauguuguguu................    5      1    Fm3
......................................................................aaguaauguaggauguugugCu................    5      1    Fm3
......................................................................aaguaUuguaggauguuguguu................    4      1    Fm3
......................................................................aaguaauguaggauguGguguu................    53     1    Fm3
......................................................................aaguaauguaggUuguuguguu................    4      1    Fm3
......................................................................aaguaauguaggauguuguCuu................    2      1    Fm3
......................................................................aaguaauguaggGuguuguguu................    1      1    Fm3
......................................................................aaguUauguaggauguuguguu................    1      1    Fm3
......................................................................aaguaaugCaggauguuguguu................    9      1    Fm3
......................................................................aaguaauguCggauguuguguu................    1      1    Fm3
......................................................................aaguaauguaggauguCguguu................    63     1    Fm3
......................................................................aaguaaCguaggauguuguguu................    2      1    Fm3
......................................................................aaguaauguaggaugCuguguu................    1      1    Fm3
......................................................................aaAuaauguaggauguuguguu................    4      1    Fm3
......................................................................aaUuaauguaggauguuguguu................    3      1    Fm3
......................................................................aaguaaAguaggauguuguguu................    2      1    Fm3
......................................................................aaguaauguaggauguugGguu................    1      1    Fm3
......................................................................aaguaauguaggaAguuguguu................    2      1    Fm3
......................................................................aaguaauguaUgauguuguguu................    22     1    Fm3
......................................................................Naguaauguaggauguuguguu................    10     1    Fm3
......................................................................aaguaauguaggauUuuguguu................    2      1    Fm3
......................................................................Gaguaauguaggauguuguguu................    4      1    Fm3
......................................................................aCguaauguaggauguuguguu................    2      1    Fm3
......................................................................aaguaauguaggaCguuguguu................    3      1    Fm3
......................................................................aaguaauguaggauguuguguu................    19072  0    Fm3
......................................................................aaguGauguaggauguuguguu................    10     1    Fm3
......................................................................aaguaauguaggaGguuguguu................    2      1    Fm3
......................................................................aaguaauguaggauguugAguu................    4      1    Fm3
......................................................................aaguaauguaggauguuUuguu................    75     1    Fm3
......................................................................aaguaaugAaggauguuguguu................    2      1    Fm3
......................................................................aGguaauguaggauguuguguu................    2      1    Fm3
......................................................................aaguaauguaggauguuCuguu................    96     1    Fm3
......................................................................aaguaauguaCgauguuguguu................    19     1    Fm3
......................................................................aaguaauCuaggauguuguguu................    1      1    Fm3
......................................................................aaguaauguaggaGguuguguuu...............    1      1    Fm3
......................................................................aaguaauguaggauguGguguuu...............    12     1    Fm3
......................................................................aaguGauguaggauguuguguuu...............    3      1    Fm3
......................................................................aaguaauguaggauguuguguuu...............    6911   0    Fm3
......................................................................aaguaauguagUauguuguguuu...............    1      1    Fm3
......................................................................aaguaauguGggauguuguguuu...............    2      1    Fm3
......................................................................aaguaaugGaggauguuguguuu...............    2      1    Fm3
......................................................................aaguaauguaggauguuUuguuu...............    25     1    Fm3
......................................................................aaguaauguaggauguuguguuC...............    14     1    Fm3
......................................................................aaguaauguaggaCguuguguuu...............    3      1    Fm3
......................................................................aaguaauguaggaugCuguguuu...............    1      1    Fm3
......................................................................aaguaauguaggauAuuguguuu...............    1      1    Fm3
......................................................................aaguaauguaggauguugCguuu...............    2      1    Fm3
......................................................................aagAaauguaggauguuguguuu...............    4      1    Fm3
......................................................................aaguaauguaggauguuguguCu...............    3      1    Fm3
......................................................................aaCuaauguaggauguuguguuu...............    2      1    Fm3
......................................................................aaguaauguaggauguuAuguuu...............    36     1    Fm3
......................................................................aaguaauguaggauguuguUuuu...............    1      1    Fm3
......................................................................aaguaauguaggauguCguguuu...............    17     1    Fm3
......................................................................aaguaauguaggauguuguguuG...............    16     1    Fm3
......................................................................aaguaNuguaggauguuguguuu...............    1      1    Fm3
......................................................................aaguaauguaggauUuuguguuu...............    1      1    Fm3
```

```
                                 Star                                 Mature
gaagguugaaggaauacgcuccuucugaugggacaugacauucugcauuacauucauugcugauaaacugaaguaauguaggauguuguguuucaugagaaaggaagg
......................................................................Uaguaauguaggauguuguguuu...............   13    1   Fm3
......................................................................Gaguaauguaggauguuguguuu...............   3     1   Fm3
......................................................................aaguaaugCaggauguuguguuu...............   1     1   Fm3
......................................................................aagGaauguaggauguuguguuu...............   1     1   Fm3
......................................................................aaAuaauguaggauguuguguuu...............   1     1   Fm3
......................................................................aaguaauguaCgauguuguguuu...............   8     1   Fm3
......................................................................aaguaauguaggauguugAguuu...............   1     1   Fm3
......................................................................aaguaauguaAgauguuguguuu...............   6     1   Fm3
......................................................................aaguaauguaggaugAuguguuu...............   2     1   Fm3
......................................................................aaguaauguaggUuguuguguuu...............   2     1   Fm3
......................................................................aaguCauguaggauguuguguuu...............   1     1   Fm3
......................................................................aGguaauguaggauguuguguuu...............   3     1   Fm3
......................................................................aaguaUuguaggauguuguguuu...............   1     1   Fm3
......................................................................aaguaauguaUgauguuguguuu...............   3     1   Fm3
......................................................................aaguaauguaggauguAguguuu...............   18    1   Fm3
......................................................................aaguaauguaggauguuCuguuu...............   41    1   Fm3
......................................................................aCguaauguaggauguuguguuu...............   1     1   Fm3
......................................................................Caguaauguaggauguuguguuu...............   1     1   Fm3
......................................................................aaguaauguaggauguuguguuA...............   704   1   Fm3
......................................................................Naguaauguaggauguuguguuu...............   2     1   Fm3
......................................................................aUguaauguaggauguuguguuu...............   6     1   Fm3
......................................................................aaguaauguaggaAguuguguuu...............   1     1   Fm3
......................................................................aaguaGuguaggauguuguguuu...............   1     1   Fm3
......................................................................aaguaauguaggauguuguguAu...............   2     1   Fm3
......................................................................aaguaauguaggauguuguguuuU..............   189   1   Fm3
......................................................................aaguaauguaggauguuguguuuA..............   1     1   Fm3
......................................................................aaguaauguaggauguuguguuuc..............   1     0   Fm3
.......................................................................aguaauguaggauguugu...................   6     0   Fm3
.......................................................................aguaauguUggauguugugu.................   1     1   Fm3
.......................................................................aguaauguaggauAuugugu.................   1     1   Fm3
.......................................................................aguaauguaggauguugugu.................   53    0   Fm3
.......................................................................aguaauguaggauUuugugu.................   1     1   Fm3
.......................................................................aAuaauguaggauguugugu.................   1     1   Fm3
.......................................................................aguaauguagAauguugugu.................   1     1   Fm3
.......................................................................aguaauguaggaugCugugu.................   1     1   Fm3
.......................................................................aguaauguaggauguuguUuu................   1     1   Fm3
.......................................................................aguaauguaggGuguuguguu................   1     1   Fm3
.......................................................................aguaauguGggauguuguguu................   1     1   Fm3
.......................................................................aguaauguaggauguuUuguu................   2     1   Fm3
.......................................................................aguaaNguaggauguuguguu................   1     1   Fm3
.......................................................................aguaauguaAgauguuguguu................   1     1   Fm3
.......................................................................aguaauUuaggauguuguguu................   1     1   Fm3
.......................................................................aguaauguaggaGguuguguu................   1     1   Fm3
.......................................................................aguaauguaggauguuguguu................   34    0   Fm3
.......................................................................aguaaAguaggauguuguguu................   1     1   Fm3
.......................................................................aguaauguaggauguuguguuA...............   1     1   Fm3
.......................................................................aguaauguaggauguuguguuu...............   5     0   Fm3
.......................................................................aguaauguaggauguuguguuuU..............   2     1   Fm3
........................................................................guaauguaggauguugugu.................   9     0   Fm3
........................................................................guaauguaggauguuguguu................   6     0   Fm3
.........................................................................uaauguaggauguugugu.................   4     0   Fm3
.........................................................................uaauguaggauguuguguu................   1     0   Fm3
..........................................................................aauguaggauguuguguu................   7     0   Fm3

.................................caugacauucugcauuacauu......................................................   10    0   Mm1
.................................caugacauucugcauuacauuc.....................................................   10    0   Mm1
.................................caugacauucugcauuacauucU....................................................   14    1   Mm1
.................................caugacauucugcauuacauuca....................................................   63    0   Mm1
.................................caugacauucugcauuaUauuca....................................................   2     1   Mm1
.................................caugacauucugcauuacauucaA...................................................   3     1   Mm1
.....................................................................Caaguaauguaggauguuguguuu...............   1     1   Mm1
......................................................................aaguaauguaggauguugu...................   41    0   Mm1
......................................................................aaguaauguaggauguGgu...................   1     1   Mm1
......................................................................aaguaauguaggauguuguA..................   1     1   Mm1
......................................................................aaguaauguaggauguugug..................   80    0   Mm1
......................................................................aaguaauguaggauguCgug..................   1     1   Mm1
......................................................................aaguaauguaggauguGgug..................   1     1   Mm1
......................................................................aaguaauguaggauguuguU..................   28    1   Mm1
......................................................................aaguaauguaggauguuCugu.................   6     1   Mm1
......................................................................aaguaauguaggauguugAgu.................   1     1   Mm1
```

Star Mature

gaagguugaaggaauacgcuccuucugauggga**caugacauucugcauuacauuca**uugcugauaaacug**aaguaauguaggauguugugu**uucaugagaaaggaagg			
....aaguaauguaggauguuUugu.................	4	1	Mm1
....Gaguaauguaggauguugugu.................	1	1	Mm1
....aaguaauguaggauguAgugu.................	1	1	Mm1
....Uaguaauguaggauguugugu.................	2	1	Mm1
....aaguaauguaggauguCgugu.................	5	1	Mm1
....aaguaauguaggauguGgugu.................	6	1	Mm1
....aaguaauguaggauguuAugu.................	9	1	Mm1
....aagCaauguaggauguugugu.................	1	1	Mm1
....aaguaauguaCgauguugugu.................	1	1	Mm1
....aaguaauguaggauguugugu.................	1181	0	Mm1
....aagAaauguaggauguugugu.................	1	1	Mm1
....aaguaauguaggauguugugG.................	33	1	Mm1
....aaguCauguaggauguugugu.................	1	1	Mm1
....aaguaauguaAgauguugugu.................	3	1	Mm1
....aaguaauguaggaugCugugu.................	1	1	Mm1
....aaguaauguaggauAuugugu.................	1	1	Mm1
....aaguaauguUggauguuguguu................	1	1	Mm1
....aaguaauguaggauguugugCu................	1	1	Mm1
....aaguaauguaggauguuguguA................	7	1	Mm1
....aaguaNuguaggauguuguguu................	1	1	Mm1
....aaguaauguaggauguuUuguu................	19	1	Mm1
....aagAaauguaggauguuguguu................	1	1	Mm1
....aaguaauguaggauguCguguu................	7	1	Mm1
....aaguaauguaggauguuCuguu................	13	1	Mm1
....aaAuaauguaggauguuguguu................	2	1	Mm1
....aaguaauguaggauCuuguguu................	1	1	Mm1
....aaguaauguaggaCguuguguu................	1	1	Mm1
....aaguaauguaggauguuAuguu................	16	1	Mm1
....aUguaauguaggauguuguguu................	5	1	Mm1
....aaguaauguaggauguuguguu................	3142	0	Mm1
....Uaguaauguaggauguuguguu................	11	1	Mm1
....aaguCauguaggauguuguguu................	1	1	Mm1
....aaguaauguaCgauguuguguu................	5	1	Mm1
....aaguaauguaggaAguuguguu................	1	1	Mm1
....aaguaauguaggauguuguguG................	2	1	Mm1
....aaguaauguaggauguugAguu................	1	1	Mm1
....aaguaauguaggauguAguguu................	7	1	Mm1
....aaguaauguaggauguGguguu................	11	1	Mm1
....aaguaauguGggauguuguguu................	1	1	Mm1
....aGguaauguaggauguuguguu................	1	1	Mm1
....aaguaauguaggaGguuguguu................	1	1	Mm1
....Caguaauguaggauguuguguu................	1	1	Mm1
....aaUuaauguaggauguuguguu................	1	1	Mm1
....aaguaauguaAgauguuguguu................	5	1	Mm1
....Naguaauguaggauguuguguu................	1	1	Mm1
....aaguaauguaggauguugCguu................	2	1	Mm1
....aaguaaAguaggauguuguguu................	1	1	Mm1
....aaguaauguaggCuguuguguu................	1	1	Mm1
....aaguaauguaUgauguuguguu................	5	1	Mm1
....aaguaauguaggauguuguguuG...............	1	1	Mm1
....aaguaauguaAgauguuguguuu...............	5	1	Mm1
....aaguaauguaggauguGguguuu...............	2	1	Mm1
....Uaguaauguaggauguuguguuu...............	3	1	Mm1
....aaguaauguaggauguuAuguuu...............	4	1	Mm1
....aaguaauguaggauguuguguuA...............	71	1	Mm1
....aagAaauguaggauguuguguuu...............	1	1	Mm1
....aUguaauguaggauguuguguuu...............	2	1	Mm1
....aaguaauguaggauguuguguuu...............	1207	0	Mm1
....aaUuaauguaggauguuguguuu...............	1	1	Mm1
....aGguaauguaggauguuguguuu...............	2	1	Mm1
....aaguaaCguaggauguuguguuu...............	1	1	Mm1
....aaguaauguaggauguuUuguuu...............	6	1	Mm1
....aaguaauguaCgauguuguguuu...............	1	1	Mm1
....aaguaauguaggauguuguguuC...............	14	1	Mm1
....aaguaauguaggauguuguUuuu...............	1	1	Mm1
....aaguaauguaggauguCguguuu...............	3	1	Mm1
....aaguaauguaggauguuCuguuu...............	8	1	Mm1
....aaguaauguaUgauguuguguuu...............	2	1	Mm1
....aaguaaugGaggauguuguguuu...............	1	1	Mm1
....aaguaauguaggauguAguguuu...............	4	1	Mm1

Star Mature

gaagguugaaggaauacgcuccuucugaugggacaugacauucugcauuacauucauugcugauaaacugaaguaauguaggauguuguguuucaugagaaaggaagg

....aCguaauguaggauguuguguuu...............	1	1	Mm1
....aaguaauguaggauguuguguuuU..............	109	1	Mm1
....aguaauguaggauguugugu.................	2	0	Mm1
....aguaauguaggauguugugG.................	1	1	Mm1
....aguaauguaggauguuguguu................	1	0	Mm1
....aguaauguaggauguuguguuu...............	8	0	Mm1
..........ggaauacgcuccuucugauggga...	1	0	Mm2
.............auacgcuccuucugauggga...	4	0	Mm2
..............uacgcuccuucugauggga...	1	0	Mm2
...............acgcuccuucugauggga...	1	0	Mm2
.................................caugacauucugcauuaca..	1	0	Mm2
.................................caugacauucugcauuacau...	1	0	Mm2
.................................caugacauucugcauuacauuc...	1	0	Mm2
.................................caugacauucugcauuacauuca..	12	0	Mm2
.................................caugacauucugcauuacauucU..	1	1	Mm2
.................................caugacauucugcauuaUauuca..	1	1	Mm2
....Caaguaauguaggauguugugu.................	1	1	Mm2
....Caaguaauguaggauguuguguu................	1	1	Mm2
....Caaguaauguaggauguuguguuu...............	1	1	Mm2
....aaguaauguaggauguuC....................	1	1	Mm2
....aaguaauguaggauguug....................	14	0	Mm2
....aaguaauguaggauguuA....................	1	1	Mm2
....aaguaauguaggauguuAu...................	1	1	Mm2
....aaguaauguaggauguCgu...................	2	1	Mm2
....Gaguaauguaggauguugu...................	1	1	Mm2
....aaguaauguaggauguugA...................	1	1	Mm2
....aaguaauguaggauguugu...................	57	0	Mm2
....aaguaauguaggauguugG...................	1	1	Mm2
....aaguaauguaggauguuguA..................	2	1	Mm2
....aaguaauguaggauguuCug..................	1	1	Mm2
....aaguaauguaggauguuguU..................	3	1	Mm2
....aaguaauguaggauguugug..................	41	0	Mm2
....aaguaauguaggauguGgug..................	1	1	Mm2
....aaguaauguaggauguuAug..................	1	1	Mm2
....aaguaauguaggauguuguUu.................	1	1	Mm2
....Gaguaauguaggauguugugu.................	3	1	Mm2
....aaguaauguaggauguugugu.................	1379	0	Mm2
....aaguaauguaggauguugugC.................	2	1	Mm2
....aaguaauguaggauguugugG.................	163	1	Mm2
....aaguaauguaAgauguugugu.................	3	1	Mm2
....aaguaNuguaggauguugugu.................	1	1	Mm2
....aaguaCuguaggauguugugu.................	1	1	Mm2
....aaguaauguaUgauguugugu.................	1	1	Mm2
....aaguaauguaggauguAgugu.................	3	1	Mm2
....Uaguaauguaggauguugugu.................	1	1	Mm2
....aaguaaugAaggauguugugu.................	1	1	Mm2
....aaguaauguaggauUuugugu.................	1	1	Mm2
....aaguaauguaggauguugugA.................	2	1	Mm2
....aaguaauguaggauguGgugu.................	3	1	Mm2
....aaguaauguaggauguuCugu.................	7	1	Mm2
....aaguaauguaCgauguugugu.................	1	1	Mm2
....aaguaauguaggaCguugugu.................	1	1	Mm2
....aaguaauguaggauguuUugu.................	6	1	Mm2
....aaguaaugCaggauguugugu.................	1	1	Mm2
....aaguaauguaggauguCgugu.................	9	1	Mm2
....aaguaauguaggauguuAugu.................	11	1	Mm2
....aagAaauguaggauguuguguu................	2	1	Mm2
....aaguaaugCaggauguuguguu................	1	1	Mm2
....aaguaauguagUauguuguguu................	1	1	Mm2
....aaguaauguaggaugCuguguu................	2	1	Mm2
....aaguaauguaggauUuuguguu................	2	1	Mm2
....aaguaauguaggaAguuguguu................	2	1	Mm2
....aaguaauguaUgauguuguguu................	3	1	Mm2
....aagCaauguaggauguuguguu................	2	1	Mm2
....aaguaauguaggauguuCuguu................	14	1	Mm2
....aaguaauguaggauguuguguu................	2771	0	Mm2
....aaguaauguaggGuguuguguu................	1	1	Mm2
....aaguaauUuaggauguuguguu................	1	1	Mm2
....aaguCauguaggauguuguguu................	1	1	Mm2

```
                                Star                                 Mature
gaaggugaaggaauacgcuccuucugaugggacaugacauucugcauuacauucauugcugauaaacugaaguaauguaggauguuguguuucaugagaaaggaagg
.....................................................................aaguaauguaggauguGguguu................   4      1      Mm2
.....................................................................aGguaauguaggauguuguguu................   3      1      Mm2
.....................................................................aaguaauguaggauguugAguu................   1      1      Mm2
.....................................................................aaguaUuguaggauguuguguu................   1      1      Mm2
.....................................................................Uaguaauguaggauguuguguu................   5      1      Mm2
.....................................................................aaguaauguaggauguuAuguu................   19     1      Mm2
.....................................................................aaguaauguaggauguuguguG................   1      1      Mm2
.....................................................................aUguaauguaggauguuguguu................   1      1      Mm2
.....................................................................aaguaauguaggauguAguguu................   10     1      Mm2
.....................................................................aaguaauguaAgauguuguguu................   8      1      Mm2
.....................................................................aaguaauguaggauguCguguu................   9      1      Mm2
.....................................................................aaguaGguaggauguuguguu................   1      1      Mm2
.....................................................................Gaguaauguaggauguuguguu................   1      1      Mm2
.....................................................................aaguaauguaggauguugGguu................   1      1      Mm2
.....................................................................aaguaaAguaggauguuguguu................   1      1      Mm2
.....................................................................aaguaauguaggauguuUuguu................   7      1      Mm2
.....................................................................aaguaauguaggauguuguguA................   3      1      Mm2
.....................................................................Naguaauguaggauguuguguu................   3      1      Mm2
.....................................................................aaguaauguaCgauguuguguu................   2      1      Mm2
.....................................................................Naguaauguaggauguuguguuu...............   1      1      Mm2
.....................................................................aaguaauguaggauguugAguuu...............   1      1      Mm2
.....................................................................Uaguaauguaggauguuguguuu...............   3      1      Mm2
.....................................................................aaguaauguaggauguuAuguuu...............   5      1      Mm2
.....................................................................aaguaaugAaggauguuguguuu...............   1      1      Mm2
.....................................................................aaguaauguaggauguAguguuu...............   7      1      Mm2
.....................................................................aaguaauguaggauguuguguAu...............   2      1      Mm2
.....................................................................aaguaauguaggauguCguguuu...............   6      1      Mm2
.....................................................................aaguaauguaggauguuguguuA...............   121    1      Mm2
.....................................................................aaguGauguaggauguuguguuu...............   2      1      Mm2
.....................................................................aaguaauguaggaAguuguguuu...............   1      1      Mm2
.....................................................................aaguaauguaggauguGguguuu...............   4      1      Mm2
.....................................................................aaguaauguaggauguuUuguuu...............   5      1      Mm2
.....................................................................aaguaauguGggauguuguguuu...............   1      1      Mm2
.....................................................................aaguaauguaUgauguuguguuu...............   1      1      Mm2
.....................................................................aaguaauguaggauguuguguuu...............   1394   0      Mm2
.....................................................................aaguaauguaggauguuguguuC...............   4      1      Mm2
.....................................................................aaguaauguaggauguuguguuG...............   8      1      Mm2
.....................................................................aaguaauguaggauguugCguuu...............   1      1      Mm2
.....................................................................aaguaauguaAgauguuguguuu...............   1      1      Mm2
.....................................................................aaguaauguaggauguuCuguuu...............   6      1      Mm2
.....................................................................aaguaauguaggauguuguguuuU..............   80     1      Mm2
.....................................................................aaguaauguaggauguuguguuuA..............   1      1      Mm2
......................................................................aguaauguaggauguugugu.................   1      0      Mm2
......................................................................aguaauguaggauguuguguu................   2      0      Mm2
......................................................................aguaauguaggauguuguguuA...............   1      1      Mm2
......................................................................aguaauguaggauguuguguuu...............   1      0      Mm2
.......................................................................guaauguaggauguuguguuu...............   1      0      Mm2
.........................................................................aauguaggauguuguguu................   2      0      Mm2

................................caugacauucugcauuacauuca....................................................   4      0      Fm1
................................caugacauucugcauuaGauuca....................................................   1      1      Fm1
.....................................................................aaguaauguaggauguuC....................   1      1      Fm1
.....................................................................aaguaauguaggauguug....................   7      0      Fm1
.....................................................................aGguaauguaggauguugu...................   2      1      Fm1
.....................................................................aaguaauguaggauguugu...................   101    0      Fm1
.....................................................................aaguaauguaggauguugG...................   1      1      Fm1
.....................................................................aaguaauguaggauguuUu...................   1      1      Fm1
.....................................................................aCguaauguaggauguugu...................   1      1      Fm1
.....................................................................aaguaauguaggauguuguU..................   6      1      Fm1
.....................................................................aaguaauguaggauguugug..................   30     0      Fm1
.....................................................................aaguaauguaggauguuguA..................   4      1      Fm1
.....................................................................aaguaauguaggauguuCugu.................   11     1      Fm1
.....................................................................aaUuaauguaggauguugugu.................   4      1      Fm1
.....................................................................Uaguaauguaggauguugugu.................   6      1      Fm1
.....................................................................aagAaauguaggauguugugu.................   2      1      Fm1
.....................................................................aaguaauguaggaugCugugu.................   1      1      Fm1
.....................................................................aaguaauguaCgauguugugu.................   4      1      Fm1
.....................................................................aaguaauguaggauAuugugu.................   1      1      Fm1
.....................................................................aaguaauguaggUuguugugu.................   1      1      Fm1
.....................................................................aaguaauguaggauguGgugu.................   5      1      Fm1
```

Star **Mature**

```
gaagguugaaggaauacgcuccuucugaugggacaugacauucugcauuacauucauugcugauaaacugaaguaauguaggauguuguguuucaugagaaaggaagg
......................................................................aaguaGuguaggauguugugu.................   1      1      Fm1
......................................................................aaguaauguaUgauguugugu.................   1      1      Fm1
......................................................................aaguaauguaggauguuAugu.................   17     1      Fm1
......................................................................aaguaauguaAgauguugugu.................   4      1      Fm1
......................................................................aaguaaCguaggauguugugu.................   2      1      Fm1
......................................................................Gaguaauguaggauguugugu.................   1      1      Fm1
......................................................................aaguaauguaggauguCgugu.................   6      1      Fm1
......................................................................aaguaauAuaggauguugugu.................   1      1      Fm1
......................................................................aaguaaAguaggauguugugu.................   1      1      Fm1
......................................................................aaguaauguaggauguuUugu.................   5      1      Fm1
......................................................................aaguaauguaggauguugugu.................   2234   0      Fm1
......................................................................aagGaauguaggauguugugu.................   2      1      Fm1
......................................................................aaguaauguaggauguugCgu.................   1      1      Fm1
......................................................................aaguaauguaggauguAgugu.................   4      1      Fm1
......................................................................aaguaauguaggauguugugG.................   447    1      Fm1
......................................................................aUguaauguaggauguugugu.................   2      1      Fm1
......................................................................aaguaauguaggauguugugA.................   1      1      Fm1
......................................................................aGguaauguaggauguuguguu................   3      1      Fm1
......................................................................aaguaauguaggauguCguguu................   13     1      Fm1
......................................................................aaguaauguaUgauguuguguu................   5      1      Fm1
......................................................................aaguaaugCaggauguuguguu................   1      1      Fm1
......................................................................aaguaauguaggauguGguguu................   8      1      Fm1
......................................................................aaguaauguaggaugGuguguu................   1      1      Fm1
......................................................................aUguaauguaggauguuguguu................   1      1      Fm1
......................................................................aaguaUuguaggauguuguguu................   1      1      Fm1
......................................................................aaguaauguaggauguugAguu................   2      1      Fm1
......................................................................Uaguaauguaggauguuguguu................   7      1      Fm1
......................................................................aaguaauguaCgauguuguguu................   1      1      Fm1
......................................................................aaguaauguaAgauguuguguu................   3      1      Fm1
......................................................................aaguaauguaggCuguuguguu................   1      1      Fm1
......................................................................aaguaauguaggGuguuguguu................   2      1      Fm1
......................................................................Gaguaauguaggauguuguguu................   4      1      Fm1
......................................................................Caguaauguaggauguuguguu................   1      1      Fm1
......................................................................aaguaauguaggaugAuguguu................   1      1      Fm1
......................................................................aagCaauguaggauguuguguu................   3      1      Fm1
......................................................................aaguaauguaggauguAguguu................   14     1      Fm1
......................................................................aaguaauguaggauguuguguG................   8      1      Fm1
......................................................................aaguaauguaggauguuCuguu................   23     1      Fm1
......................................................................aaguaauguGggauguuguguu................   1      1      Fm1
......................................................................aaguaauguaggauguuUuguu................   14     1      Fm1
......................................................................aaguaauguaggauguugugGu................   1      1      Fm1
......................................................................aaguaauguaggauguuAuguu................   25     1      Fm1
......................................................................aaguaauguaggauguugugAu................   1      1      Fm1
......................................................................aagAaauguaggauguuguguu................   1      1      Fm1
......................................................................aaguaauguaggauguuguguu................   4052   0      Fm1
......................................................................aaUuaauguaggauguuguguu................   1      1      Fm1
......................................................................aaguaauguaggauAuuguguu................   1      1      Fm1
......................................................................aaguaNuguaggauguuguguu................   1      1      Fm1
......................................................................aaguaaugAaggauguuguguu................   2      1      Fm1
......................................................................aaguaauguaggauguuguAuu................   1      1      Fm1
......................................................................aaAuaauguaggauguuguguu................   2      1      Fm1
......................................................................aaguaauguagUauguuguguu................   2      1      Fm1
......................................................................aaguaauguaggauguuAuguuu...............   15     1      Fm1
......................................................................aaguaauUuaggauguuguguuu...............   1      1      Fm1
......................................................................aaCuaauguaggauguuguguuu...............   1      1      Fm1
......................................................................aaguaauguaggauguuCuguuu...............   15     1      Fm1
......................................................................aaguaauguaggauguCguguuu...............   12     1      Fm1
......................................................................aaguaauAuaggauguuguguuu...............   1      1      Fm1
......................................................................Caguaauguaggauguuguguuu...............   1      1      Fm1
......................................................................aaguaauguaCgauguuguguuu...............   2      1      Fm1
......................................................................Gaguaauguaggauguuguguuu...............   1      1      Fm1
......................................................................aaguaauguaggauguugugCuu...............   1      1      Fm1
......................................................................aaguaNuguaggauguuguguuu...............   1      1      Fm1
......................................................................aaguaauguCggauguuguguuu...............   1      1      Fm1
......................................................................aaguaauguaggauguGguguuu...............   4      1      Fm1
......................................................................aaguaauguaggauguAguguuu...............   5      1      Fm1
......................................................................aaguaauguaggauguugugAuu...............   2      1      Fm1
......................................................................aaguaauguaUgauguuguguuu...............   2      1      Fm1
......................................................................aaguaauguaggauguuguguuu...............   3528   0      Fm1
......................................................................aaguaauguaggauguuguguuG...............   9      1      Fm1
```

Star Mature

gaagguugaaggaauacgcuccuucugaugggacaugacauucugcauuacauucauugcugauaaacugaaguaauguaggauguuguguuucaugagaaaggaagg			
....aaguaauguaggauguuguguuC...............	1	1	Fm1
....aaguaaugAaggauguuguguuu...............	1	1	Fm1
....aaguaauguaggaugAuguguuu...............	1	1	Fm1
....aaguaauguaggGuguuguguuu...............	1	1	Fm1
....aaguaauguaggauguuguguGu...............	1	1	Fm1
....Naguaauguaggauguuguguuu...............	2	1	Fm1
....aagAaauguaggauguuguguuu...............	4	1	Fm1
....aaguaauguaggauguugCguuu...............	1	1	Fm1
....Uaguaauguaggauguuguguuu...............	5	1	Fm1
....aaguaauguaAgauguuguguuu...............	5	1	Fm1
....aaguaauguaggauguuUuguuu...............	10	1	Fm1
....aaguaauCuaggauguuguguuu...............	1	1	Fm1
....aGguaauguaggauguuguguuu...............	3	1	Fm1
....aaguaauguaggauguuguguuA...............	223	1	Fm1
....aaguaUuguaggauguuguguuu...............	1	1	Fm1
....aagGaauguaggauguuguguuu...............	2	1	Fm1
....aagCaauguaggauguuguguuu...............	1	1	Fm1
....aaguaauguaggUuguuguguuu...............	1	1	Fm1
....aaguaauguaggauguuguguuuU..............	109	1	Fm1
....aaguaauguaggauguCguguuuc..............	1	1	Fm1
....aaguaauguaggauguuguguuuc..............	1	0	Fm1
....aguaauguaggauguugu...................	1	0	Fm1
....aguaaAguaggauguugugu.................	1	1	Fm1
....aguaauguaggauguugugu.................	1	0	Fm1
....aguaauguaggauguuguguu................	12	0	Fm1
....aguaauguaggauguuguguuu...............	1	0	Fm1
....Caaguaauguaggauguuguguu................	5	1	Fm2
....aaguaauguaggauguug....................	6	0	Fm2
....aaguaauguaggauguugu...................	15	0	Fm2
....aaguaauguaggauguuCu...................	1	1	Fm2
....aaguaauguaggauguugug..................	5	0	Fm2
....aaguaauguaggauguuguA..................	3	1	Fm2
....Caguaauguaggauguugugu.................	1	1	Fm2
....aaguaauguaggauguAgugu.................	2	1	Fm2
....aaguaauguaggauguugugG.................	51	1	Fm2
....aaguaauguaggauguuAugu.................	1	1	Fm2
....aaguaauguaggauguugugu.................	339	0	Fm2
....aGguaauguaggauguugugu.................	1	1	Fm2
....aaguaauguaggauguuUugu.................	2	1	Fm2
....aaguaauguaggauguuguguG................	4	1	Fm2
....aaguaauguaggauguuAuguu................	16	1	Fm2
....aaguaauguaggauguuCuguu................	26	1	Fm2
....aaguaauguCggauguuguguu................	1	1	Fm2
....aaguaauguaggauguAguguu................	11	1	Fm2
....aaguaauguaggauguGguguu................	7	1	Fm2
....aaguaauguaggUuguuguguu................	1	1	Fm2
....aaguaauguaggauguuUuguu................	23	1	Fm2
....aUguaauguaggauguuguguu................	1	1	Fm2
....aaguaauguaggauguCguguu................	13	1	Fm2
....aaguaaCguaggauguuguguu................	1	1	Fm2
....aaguaauguaggauguuguAuu................	1	1	Fm2
....aaguaauguGggauguuguguu................	2	1	Fm2
....aaguaauguUggauguuguguu................	1	1	Fm2
....aaAuaauguaggauguuguguu................	1	1	Fm2
....Naguaauguaggauguuguguu................	4	1	Fm2
....aaguaauguaUgauguuguguu................	8	1	Fm2
....aGguaauguaggauguuguguu................	2	1	Fm2
....aaCuaauguaggauguuguguu................	1	1	Fm2
....Gaguaauguaggauguuguguu................	1	1	Fm2
....aaguaauUuaggauguuguguu................	1	1	Fm2
....aaguaNuguaggauguuguguu................	1	1	Fm2
....aaguaauguaggaAguuguguu................	1	1	Fm2
....aaguaauguaggaGguuguguu................	1	1	Fm2
....aaguaauguaAgauguuguguu................	6	1	Fm2
....aaguaauguaggauguuguUuu................	1	1	Fm2
....aaguUauguaggauguuguguu................	1	1	Fm2
....aaguaauguagUauguuguguu................	1	1	Fm2
....aaguGauguaggauguuguguu................	1	1	Fm2
....aCguaauguaggauguuguguu................	1	1	Fm2

Star Mature

```
gaagguugaaggaauacgcuccuucugaugggacaugacauucugcauuacauucauugcugauaaacugaaguaauguaggauguuguguuucaugagaaaggaagg
......................................................................aaguaauguaCgauguuguguu................	5	1	Fm2
......................................................................aaguaauguaggauguuguguu................	4102	0	Fm2
......................................................................aaguaauguaggGuguuguguu................	2	1	Fm2
......................................................................Uaguaauguaggauguuguguu................	17	1	Fm2
......................................................................aaguaaCguaggauguuguguuu...............	1	1	Fm2
......................................................................aaguaauguaggauguCguguuu...............	3	1	Fm2
......................................................................Uaguaauguaggauguuguguuu...............	1	1	Fm2
......................................................................aaguaauguaggauguuguguuA...............	183	1	Fm2
......................................................................aaguaauguaggauguuAuguuu...............	8	1	Fm2
......................................................................aagCaauguaggauguuguguuu...............	1	1	Fm2
......................................................................aaguaauguaggauAuuguguuu...............	1	1	Fm2
......................................................................aaguaauguaggauguuguguuC...............	20	1	Fm2
......................................................................aUguaauguaggauguuguguuu...............	2	1	Fm2
......................................................................aaguaauguaAgauguuguguuu...............	2	1	Fm2
......................................................................aaguaauguaggauguuguguuu...............	1864	0	Fm2
......................................................................aaguaauguaggauguAguguuu...............	5	1	Fm2
......................................................................aaAuaauguaggauguuguguuu...............	1	1	Fm2
......................................................................aaguaauguaggauguuguguuG...............	15	1	Fm2
......................................................................aaguaauguaggaAguuguguuu...............	1	1	Fm2
......................................................................aaguaauguaggauguugugAuu...............	1	1	Fm2
......................................................................aagGaauguaggauguuguguuu...............	1	1	Fm2
......................................................................aaguaUuguaggauguuguguuu...............	1	1	Fm2
......................................................................aaguaauguaggauguuguCuuu...............	1	1	Fm2
......................................................................aaguaauguaggauguuCuguuu...............	9	1	Fm2
......................................................................aaguaaugCaggauguuguguuu...............	1	1	Fm2
......................................................................aaguaauguaggauguGguguuu...............	8	1	Fm2
......................................................................aaguaauguaggauguuUguuu...............	7	1	Fm2
......................................................................aaguaauguaCgauguuguguuu...............	2	1	Fm2
......................................................................aaguaauguaggauguuguguuuU..............	114	1	Fm2
.......................................................................aguaauguaggauUuuguguu................	1	1	Fm2
.......................................................................aguaauguaggauguuguguu................	1	0	Fm2
.......................................................................aguaauguaggauguuguguuA...............	5	1	Fm2
```
